# Supplementary material for: Introduction of a Post-Anaesthesia Care Unit in a Teaching Hospital Is Associated with a Reduced Length of Hospital Stay in Noncardiac Surgery: A Single-Centre Interrupted Time Series Analysis
Source: J Clin Med. 2024 Jan 17;13(2):534. doi: 10.3390/jcm13020534 (PMC10816897; doi:10.3390/jcm13020534)
Supplement: Supplementary file 1 [file jcm-13-00534-s001.zip › jcm-2805551-supplementary.pdf]

Supplemental Section S1

Data extraction for pre-existing conditions and postoperative complications

All of the patients' diagnoses (Diagnosis Related Group Codes) registered in the EHR were extracted and divided into preoperatively and postoperatively registered diagnoses for detection of all of the pre-existing conditions and postoperative complications of interest. Only new postoperative diagnoses registered within 30 days after surgery were reviewed. All medication use was extracted and divided into preoperative and new postoperative prescriptions. Moreover, the number of different medications was based on the amount of different Anatomical Therapeutic Chemical codes preoperatively per patient.

Laboratory data extraction included preoperative levels of haemoglobin, estimated glomerular filtration rate (eGFR) and NT-pro-BNP and postoperative eGFR, NT-pro-BNP and high-sensitive Troponin I or T.

The presence of the following Dutch Healthcare Authority procedural codes (NZa activity codes) was extracted for the current cohort in the postoperative phase until 30 days postoperatively: 33229 (Cardiac catheterization left and/or right-sided, including angiography), 33231 (percutaneous transluminal coronary angioplasty for treatment of stenosis of coronary arteries), 33232 (percutaneous transluminal coronary angioplasty for treatment of stenosis of multiple coronary arteries or left main coronary artery), 33233 (percutaneous transluminal coronary angioplasty for treatment of chronic occlusion of coronary artery), 33234 (percutaneous transluminal coronary angioplasty of stenosis of coronary artery bypass graft), 33238 (acute percutaneous transluminal coronary angioplasty for treatment of stenosis of coronary arteries), 39476 (fractional flow reserve), 39493 (transthoracic echocardiography), 39494 (transoesophageal echocardiography), 39757 (review of ECG, Holter monitor or cardiac stress test), 39852 (Acute non-invasive ventilation), 190015 (Emergency room visit), 190129 (Intensive Care Unit consultation), 190158 (Intensive Care Unit admission).

All ICU admissions, readmissions or reoperations within 30 days of the initial surgery were collected. Moreover, length of hospital stay, duration of stay at IMCU or PACU and postoperative mortality up to 1 year were recorded.

Extraction of pre-existing conditions and postoperative complications from EHR

Pre-existing conditions and postoperative complications were registered when one of the following criteria were present or led to a manual chart review to check for the presence or absence of relevant conditions or events. Moreover, all cases of postoperative ICU admission or mortality were reviewed for the presence of postoperative complications.

Pre-existing conditions (registered before date of surgery)

- Coronary artery disease
  - Diagnosis of myocardial infarction, myocardial ischemia, ischemic cardiomyopathy or follow-up following CABG or coronary intervention
  - Use of ticagrelor or sublingual nitrates led to manual chart review
- Congestive heart failure
  - Diagnosis of systolic or diastolic heart failure, history of decompensated heart failure
  - Elevated NT-pro-BNP above 100 pg ml^{-1} led to manual chart review.
- Valvular heart disease
  - Diagnosis of any valvular insufficiency or stenosis or follow-up following valve replacement
- Atrial fibrillation
  - Diagnosis of atrial fibrillation or paroxysmal atrial fibrillation
- Insulin-dependent diabetes mellitus
  - Diagnosis of diabetes mellitus type 1 or type 2 with insulin dependency
  - Registration of insulin use in medication section

- COPD
  - Diagnosis of COPD, emphysema, chronic bronchitis or history of COPD exacerbation
- Respiratory insufficiency other than COPD
  - Diagnosis of asthma, interstitial lung disease, bronchiectasis or chronic respiratory insufficiency.
- Chronic renal failure
  - Diagnosis of chronic renal failure, presence or history of renal transplant, history of an arteriovenous shunt surgery,
  - Estimated glomerular filtration rate below 60 ml min^{-1} per 1.73 m^2 led to manual chart review
  - Registration of haemodialysis or peritoneal dialysis in the medication section
  - Use of phosphate binders led to manual chart review
- Stroke
  - Diagnosis of ischaemic or haemorrhagic stroke, transient ischemic attack or vascular dementia.
- Anaemia
  - Diagnosis of anaemia within one year preoperatively.
  - Preoperative haemoglobin was below 13.0 g dl^{-1} (male) or 12.0 g dl^{-1} (female) within 1 month preoperatively.

Postoperative complications (registered postoperatively until 30 days after surgery)

- Myocardial ischaemia
  - New diagnosis of myocardial infarction or myocardial ischemia
  - Elevated troponin I or T led to manual chart review
  - Presence of NZa activity code associated with possible myocardial ischemia led to manual chart review: 33229 (Cardiac catheterization left and/or right-sided, including angiography), 33231 (percutaneous transluminal coronary

angioplasty for treatment of stenosis of coronary arteries), 33232 (percutaneous transluminal coronary angioplasty for treatment of stenosis of multiple coronary arteries or left main coronary artery), 33233 (percutaneous transluminal coronary angioplasty for treatment of chronic occlusion of coronary artery), 33234 (percutaneous transluminal coronary angioplasty of stenosis of coronary artery bypass graft), 33238 (acute percutaneous transluminal coronary angioplasty for treatment of stenosis of coronary arteries), 39476 (fractional flow reserve), 39493 (transthoracic echocardiography), 39494 (transoesophageal echocardiography), 39757 (review of ECG, Holter monitor or cardiac stress test), 39852 (Acute non-invasive ventilation), 190015 (Emergency room visit), 190129 (Intensive Care Unit consultation), 190158 (Intensive Care Unit admission).

- Decompensated heart failure
    - New diagnosis of decompensated heart failure
    - Elevated NT-pro-BNP above 100 pg ml^{-1} led to manual chart review.
    - Presence of activity code associated with possible decompensated heart failure led to manual chart review: 39493 (transthoracic echocardiography), 39494 (transoesophageal echocardiography), 39757 (review of ECG, Holter monitor or cardiac stress test), 39852 (Acute non-invasive ventilation), 190015 (Emergency room visit), 190129 (Intensive Care Unit consultation), 190158 (Intensive Care Unit admission).
- New-onset atrial fibrillation
    - New diagnosis of atrial fibrillation
    - Presence of activity code associated with possible new-onset atrial fibrillation led to manual chart review: 39493 (transthoracic echocardiography), 39494 (transoesophageal echocardiography), 39757 (review of ECG, Holter monitor or cardiac stress test).

- Postoperative Pulmonary Complications
    - New diagnosis of pneumonia, atelectasis, respiratory insufficiency, pneumothorax, exacerbation of COPD or asthma.
    - Presence of activity code associated with possible postoperative pulmonary complication led to manual chart review: 39852 (Acute non-invasive ventilation), 190015 (Emergency room visit), 190129 (Intensive Care Unit consultation) or 190158 (Intensive Care Unit admission).
- Stroke
    - New diagnosis of ischemic or haemorrhagic stroke or transient ischemic attack.
    - Presence of activity code associated with possible stroke led to manual chart review: 190015 (Emergency room visit), 190129 (Intensive Care Unit consultation) or 190158 (Intensive Care Unit admission).
- Acute Kidney Injury
    - New diagnosis of acute renal failure or acute kidney injury
    - New diagnosis of chronic renal failure led to manual chart review
    - Presence of activity code associated with possible AKI led to manual chart review: 190129 (Intensive Care Unit consultation) or 190158 (Intensive Care Unit admission).
    - Decrease of postoperative to preoperative estimated glomerular filtration rate of 25% or more.

- Need for Renal Replacement Therapy:
    - New registration in medication section of haemodialysis
    - New diagnosis of acute renal failure, acute kidney injury or chronic renal failure led to manual chart review
    - Presence of activity code associated with possible RRT led to manual chart review: 190129 (Intensive Care Unit consultation) or 190158 (Intensive Care Unit admission).
- Pulmonary embolism
    - New diagnosis of pulmonary embolism
    - Presence of activity code associated with possible pulmonary embolism led to manual chart review: 39493 (transthoracic echocardiography), 39494 (transoesophageal echocardiography), 39757 (review of ECG, Holter monitor or cardiac stress test), 190015 (Emergency room visit), 190129 (Intensive Care Unit consultation) or 190158 (Intensive Care Unit admission).

Supplemental Section S2

Patient outcome for the fraction of patients admitted to the Intermediate Care Unit or Post Anaesthesia Care Unit

	**InterMediate Care Unit episode (n=678)**	**Post Anaesthesia Care Unit episode (n=625)**	**P-value**
**Any postoperative complication**	100 (14.7%)	83 (13.3%)	0.473
Myocardial ischemia	19 (2.8%)	11 (1.8%)	0.268
Decompensated heart failure	23 (3.4%)	13 (2.1%)	0.177
New-onset atrial fibrillation	20 (2.9%)	30 (4.8%)	0.085
PPC	44 (6.5%)	31 (5.0%)	0.284
Stroke	4 (0.6%)	9 (1.4%)	0.164
AKI	30 (4.4%)	15 (2.4%)	0.049
Need for RRT	1 (0.1%)	1 (0.2%)	1.000
Pulmonary embolism	7 (1.0%)	6 (1.0%)	1.000
**ICU admission**	52 (7.7%)	43 (6.9%)	0.596
**ICU admission for medical indication**	50 (7.4%)	29 (4.6%)	0.048
**Reoperation**	69 (10.2%)	54 (8.6%)	0.393
**Readmission**	110 (16.2%)	90 (14.4%)	0.398
**Mortality at 30 days**	30 (4.4%)	20 (3.2%)	0.312
**Mortality at 3 months**	62 (9.1%)	38 (6.1%)	0.047
**Mortality at 1 year**	110 (16.2%)	74 (11.8%)	0.026

AKI = acute kidney injury, ICU = intensive care unit, PPC = postoperative pulmonary complications, RRT = renal replacement therapy.
